# Advances in Optical Adjunctive Aids for Visualisation and Detection of Oral Malignant and Potentially Malignant Lesions

**DOI:** 10.1155/2013/194029

**Published:** 2013-09-02

**Authors:** Nirav Bhatia, Yastira Lalla, An N. Vu, Camile S. Farah

**Affiliations:** ^1^School of Dentistry, The University of Queensland, Brisbane, QLD 4000, Australia; ^2^UQ Centre for Clinical Research, Royal Brisbane & Women's Hospital, The University of Queensland, Herston, QLD 4029, Australia

## Abstract

Traditional methods of screening for oral potentially malignant disorders and oral malignancies involve a conventional oral examination with digital palpation. Evidence indicates that conventional examination is a poor discriminator of oral mucosal lesions. A number of optical aids have been developed to assist the clinician to detect oral mucosal abnormalities and to differentiate benign lesions from sinister pathology. This paper discusses advances in optical technologies designed for the detection of oral mucosal abnormalities. The literature regarding such devices, VELscope and Identafi, is critically analysed, and the novel use of Narrow Band Imaging within the oral cavity is also discussed. Optical aids are effective in assisting with the detection of oral mucosal abnormalities; however, further research is required to evaluate the usefulness of these devices in differentiating benign lesions from potentially malignant and malignant lesions.

## 1. Introduction

Oral cancer affects the lips, tongue, gingiva, floor of mouth, palate, tonsils, and oropharynx [1–3]. It is ranked the sixth most common malignancy worldwide and is diagnosed at an increasing rate [4], with an estimated 263,900 new cases and 128,000 deaths in 2008 alone [5]. Oral squamous cell carcinoma (OSCC) can affect any tissue lined with oral mucosal epithelium and accounts for 90% of oral malignancies [1, 4]. Known aetiological risk factors for OSCC include tobacco, betel quid, alcohol, and micronutrient deficiency [2, 6, 7]; however, recent studies also implicate human papillomavirus (HPV) as a causative factor in cancers of the base of the tongue, tonsils, and oropharynx in patients without traditional risk factors [1, 2, 7].

Despite advances in cancer therapies, the five-year survival rate for oral cancer has remained at approximately 50% over the past three decades [4, 8]. This is primarily due to delayed diagnosis, with approximately half of all oral cancers diagnosed at stages III or IV [9]. By these stages, lymphatic spread has occurred and treatment is for a systemic condition rather than a localized disease process. Localised cancers have survival rates of up to 83% but this falls to 32% once tumour metastasis has occurred [10]. As such, emphasis should be placed on earlier detection of oral cancers to improve patient survival rates.

OSCC is often preceded by visible and histological changes in the oral mucosa. Conditions which have the potential to develop into malignancies are referred to as oral potentially malignant disorders (OPMDs) and these include leukoplakia, erythroplakia, oral submucous fibrosis, oral lichen planus, and actinic keratosis [11]. Although only a small proportion of OPMDs undergo malignant transformation, the key to improved patient prognosis is believed to be through early detection and management of these lesions [12, 13].

The current protocol for detecting OPMDs by conventional oral examination (COE) involves visual inspection of the oral cavity and tactile examination of head and neck lymph nodes by a medical or dental practitioner. However, even with meticulous follow up, early malignant changes are still overlooked using COE [14] as dysplasia may be found in clinically normal mucosa [15, 16]. While a recent meta-analysis reported 93% sensitivity for COE, specificity was poor at only 31%. Therefore, COE cannot reliably differentiate between benign and dysplastic lesions, and this is most likely due to the fact that a number of benign conditions mimic oral malignancies [15]. Epstein et al. [15] suggested that further research into adjunct visualisation technologies is required to improve the reliability of clinicians in screening for malignant and potentially malignant disorders.

A variety of devices utilising the principles of tissue autofluorescence, tissue reflectance, or narrow band imaging (NBI) have been commercialised as adjunctive aids to COE for the detection of OPMDs and OSCCs. This paper critically appraises the literature regarding commercially available devices VELscope, Identafi, and Narrow Band Imaging, and discusses their application in the oral cavity, as well as highlighting other approaches to optical imaging.

## 2. VELscope

Autofluorescence is a phenomenon whereby an extrinsic light source is used to excite endogenous fluorophores such as certain amino acids, metabolic products, and structural proteins [26]. Within the oral mucosa, the most relevant fluorophores are nicotinamide adenine dinucleotide (NADH) and flavin adenine dinucleotide (FAD) in the epithelium and collagen cross-links in the stroma [27]. The fluorophores absorb photons from the exogenous light source and emit lower energy photons which present clinically as fluorescence [28]. Each fluorophore is associated with specific excitation and emission wavelengths.

Mucosal abnormalities can alter the absorption and scattering properties of tissue due to changes in tissue architecture and concentrations of fluorophores. *In vitro *studies have shown a decrease in autofluorescence in oral epithelial dysplasia (OED) as well as mucosal inflammation [27, 29]. Multiple oncological applications for *in vivo* fluorescence spectroscopy have previously been described [30]. Preliminary research indicates that autofluorescence is a suitable adjunct to COE in early detection of OSCC and OPMD [28, 31–34]. 

VELscope (LED Medical Diagnostics Inc., Barnaby Canada) utilises blue light excitation between 400 and 460 nm wavelength [1, 35–37] to enhance oral mucosal abnormalities by direct tissue autofluorescence. At these excitation wavelengths, normal oral mucosa is associated with a pale green fluorescence when viewed through a filter, whereas abnormal tissue is associated with a loss of autofluorescence (LAF) and appears dark [1]. Although pilot studies found that these excitation wavelengths could be used *in vivo *to differentiate normal oral mucosa from dysplasia, carcinoma in situ (CIS), and invasive carcinoma, the manufacturer extrapolated these findings to indicate that VELscope can help detect oral mucosal abnormalities not visible under white light examination [33, 38].

Early research supporting the use of VELscope is comprised of case reports regarding its use on referred or review patients at specialist oral dysplasia clinics [17, 39]. Kois and Truelove [39] found that VELscope assisted in the detection of dysplastic and malignant lesions not visible by COE and helped raise suspicion of lesions which would otherwise not be subjected to biopsy. In one particular case where widespread erythema was present, VELscope revealed an area which later proved to be a well-differentiated carcinoma. It also demonstrated its value in demarcating margins of established tumours where the malignant tissue extended beyond what was otherwise clinically visible [25]. While the use of VELscope was in specialist environments, these case reports provided initial evidence that the device enabled clinicians to differentiate dysplasia from normal oral mucosa.

The diagnostic accuracy of VELscope in detecting dysplasia and OSCC has been studied extensively in specialist referral centres [18–20, 22, 24, 40], with reported sensitivities ranging from 30 to 100% (Table 1) [18–20, 22, 24, 40]. Despite the large range, some studies noted that VELscope helped discover dysplastic lesions missed by COE [19, 40]. For these reasons, VELscope appears to be a valuable tool in monitoring patients with a history of head and neck cancer. However, Mehrotra et al. [22] argued that since not all dysplastic lesions displayed LAF, its use in routine practice should be discouraged as it can result in missed lesions and a false sense of security. Of concern, VELscope has a fairly high rate of false positives, with reported specificities ranging between 15 to 81% (Table 1) [18–20, 22, 24, 40]. This suggests that VELscope is a poor differentiator between benign and dysplastic lesions [18, 20]. In particular, inflammatory lesions typically display LAF as well and, thus, act as confounders when using VELscope [19]. As the majority of oral mucosal lesions seen in general practice are benign in nature, incorrect interpretation can lead to overestimation of oral mucosal abnormalities and patient harm through unnecessary referrals and biopsies.

**Figure 1:**
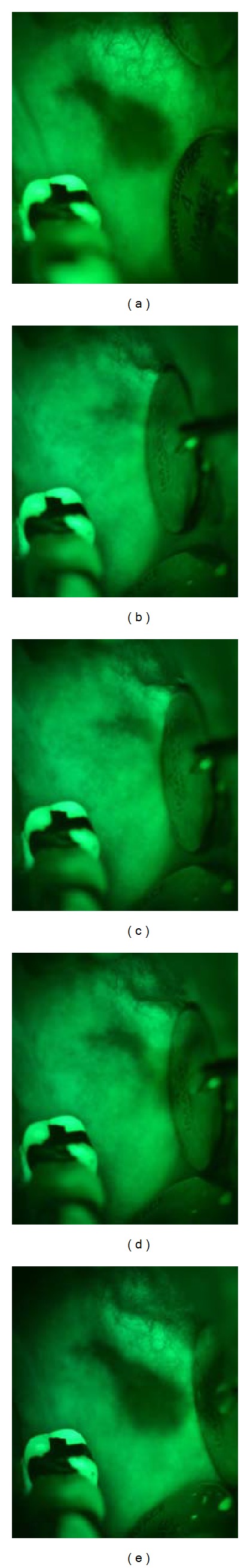
Oral lichen planus on left buccal mucosa displaying loss of autofluorescence when visualised using VELscope (a). The same lesion displaying diascopic fluorescence on application of pressure (b)–(d), returning to its original appearance when pressure is removed (e).

While the majority of studies have evaluated VELscope's diagnostic capabilities and accuracy without taking into account clinical characteristics, VELscope's main function is to serve as an adjunctive aid to rather than a replacement for COE. For this reason, Farah et al. [19] prospectively evaluated the use of VELscope in conjunction with COE in a specialist environment. Lesions which displayed diascopic fluorescence were considered negative for LAF. Sensitivity was higher when VELscope and COE findings were combined than for either COE or VELscope alone, whereas specificity only increased slightly [19]. This highlights the importance of clinical interpretation when using VELscope rather than relying on LAF on its own. The authors also assessed the effect of diascopic fluorescence, whereby lesions displaying LAF return to a normal fluorescence pattern with the application of pressure (Figure 1). This technique enables clinicians to differentiate inflammatory lesions from neoplasia since inflammatory lesions typically display complete diascopic fluorescence, whereas neoplastic lesions do not [19]. These results cannot be generalised to general practitioners as the study was performed by specialists, and advanced knowledge of mucosal pathology is beneficial to effectively differentiate between LAF and diminished autofluorescence [19, 20, 24]. Furthermore, Farah et al. [19] observed that complete blanching of lesions was difficult to achieve and partial blanching could complicate interpretation. Therefore, VELscope is vulnerable to interoperator variability [24].

Thus far, research on the use of VELscope for routine screening in the general population is limited (Table 2). Huff et al. [41] found an increased rate of detection of OED using VELscope in a private practice setting when compared to COE. However, this was conducted in parallel cohorts and the clinical characteristics of lesions discovered with VELscope were not discussed. It is unclear whether VELscope aided the detection of new lesions or helped raise the suspicion of lesions detected by COE. Nonetheless, similar results were found during routine screening of patients attending student clinics at a dental school [42]. McNamara et al. [43], however, found the low specificity of VELscope to be a barrier for its use in routine screening in general practice, arguing that it would lead to a large number of overreferrals. In their protocol, the authors did not consider VELscope findings in context by reexamining areas with LAF clinically, despite the fact that LAF alone has little meaning without assessing the site again by COE to eliminate contribution of inflammatory, pigmented, or vascular lesions to this phenomenon. In addition, a case of moderate dysplasia of the lip did not display LAF, repeating concerns that dysplastic lesions may be missed. Future studies assessing the efficacy of VELscope in routine practice should consider both clinical and VELscope findings to assess how these collectively impact on the specificity of the device. Consideration of a tested algorithm and decision making protocol would be helpful for general practitioners utilising the device. 

The existing literature indicates that VELscope can differentiate between normal mucosa and mucosal abnormalities; however, it is not highly specific in detecting OPMDs and as a result gives rise to a high rate of false positives. The sensitivity varies among studies (Table 1) and this could be due to interoperator variability in what constitutes LAF. It has been reported that there is a large spectrum of fluorescence intensity and a more definitive criteria of what constitutes LAF are required to reduce subjectivity and support the use of the device in wider clinical practice [24]. Furthermore, it has been suggested that a significant understanding of mucosal pathology is required to make correct clinical interpretations of VELscope findings [19], and this understanding may not be present in a general practice environment. Future research directions should evaluate the biological bases that contribute to false-positive and false-negative findings. If the specificity of the device could be improved, there would be an increased scope for the use of VELscope in routine general practice.

## 3. Identafi

The Identafi (DentalEZ, PA, USA) is a multispectral screening device that incorporates three different lights which are designed to be used in a sequential manner to facilitate intraoral examination [44, 45]. In addition to a LED white light, Identafi also includes violet and green-amber lights to induce direct tissue fluorescence and tissue reflectance, respectively. Although previous research found that white light allows superior visualisation of oral mucosal lesions compared to routine incandescent light [46], differentiating between OPMDs and normal mucosa is still difficult with white light alone. By integrating tissue fluorescence and tissue reflectance into the one device, Identafi aims to be an easy to use device maximising the advantages of both white light examination and tissue fluorescence. 

**Figure 2:**
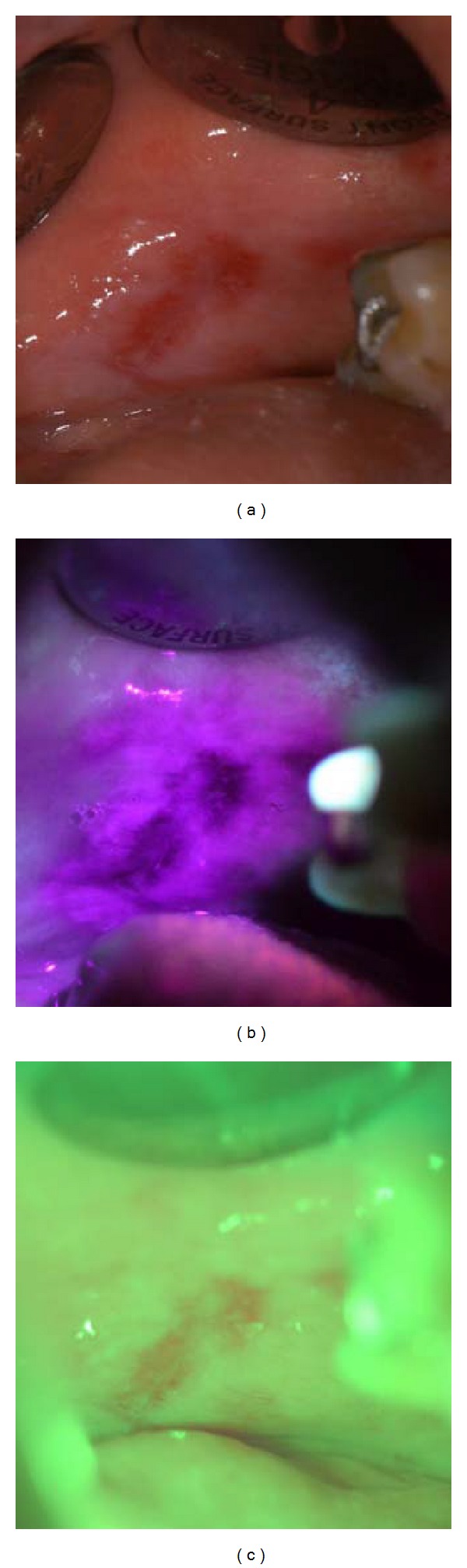
Oral lichen planus on left buccal mucosa visualised with Identafi using its white light feature (a). The same lesion displaying loss of autofluorescence when visualised under violet light with Identafi (b), and microvasculature of the lesion is highlighted with the green-amber light (c).

Violet light 405 nm in wavelength is used to assess, through the accompanying photosensitive filter glasses, the autofluorescence properties of oral tissues. As with VELscope, normal mucosa exhibits natural fluorescence, whereas abnormal tissues appear dark due to diminished autofluorescence or LAF (Figure 2). A study by Roblyer et al. [28] reported that light at 405 nm was the optimal excitation wavelength for discriminating between normal oral mucosa and dysplasia or OSCC, as it had 96 to 100% sensitivity and 91 to 96% specificity. However, a study by Sweeny et al. [47] involving 88 patients with a history of head and neck cancer reported 50% sensitivity and 81% specificity for Identafi's violet light and 50% sensitivity and 98% specificity for COE. The authors suggested radiation-induced changes such as fibrosis and pigmentation as possible causes for the low sensitivity. Another contributing factor was the lack of histopathology, as biopsies were not taken for every lesion, so it is possible that some areas with LAF had underlying dysplasia which was not apparent clinically. Results from this study must also be interpreted with caution as there was no indication whether or not OED, OSCC, or both were considered as positive findings. Nonetheless, preliminary cases by Lane et al. [21, 48] noted that areas of LAF were often larger than the clinically visible cancer when observed with violet light. They attributed this to the visualisation of deeper neovascularisation and stromal changes which accompany lesion progression, and therefore proposed that this technology could assist in determining surgical margins for the excision of lesions.

The green-amber light at 545 nm wavelength utilises the concept of reflectance spectroscopy to delineate the vasculature in the connective tissue (Figure 2). Reflectance spectroscopy uses light within the absorption spectrum of haemoglobin—namely, between 400 and 600 nm—to visualise the underlying vasculature [49]. A significant reduction in the reflectance spectra of OSCCs and OPMDs occurs at 577 nm and 542 nm, and this is attributed to increased light absorption from increased microvasculature density and oxygenated haemoglobin concentrations in neoplastic tissue. Angiogenesis is an early step in carcinogenesis and a significant increase in microvessel count occurs in mild and moderate dysplasia [50–52]. Existing evidence also indicates that tumour-induced angiogenesis results in altered vascular morphology, and the degree of change can assist with determining the prognosis of oral lesions [50, 51]. This suggests that assessment of tissue angiogenesis in oral mucosal lesions enables the clinician to differentiate OPMDs from benign lesions. To date, however, there are very few published reports regarding the use of the 545 nm green-amber light. While the green-amber light was effective for highlighting the superficial vasculature and enhancing the keratinization of lesions in one study [21], another study [47] reported 0% sensitivity as no true-positive findings were detected. However, the authors acknowledged the low power of the study and that further research was required.

The publications thus far place little emphasis on the importance of the white light function of the device, and at this stage there is limited evidence to suggest any advantage of Identafi over COE. Results cannot be generalised to general practitioners as screening clinicians in these studies had specialist level training [21, 47]. Furthermore, there are currently no published clinical trials on the routine use of Identafi in the general population which were not funded by the manufacturer of the device. The ability for Identafi to differentiate between low- and high-risk lesions remains undetermined with the existing literature. There are currently several ongoing clinical trials [53], including several in our group, and until complete results from further research is published, use of the Identafi as a visualisation adjunct for OPMDs and OSCCs can only be justified based on our knowledge of comparable optical fluorescence imaging devices.

## 4. Narrow Band Imaging 

Narrow band imaging (NBI; Olympus Medical Systems Corporation, Tokyo, Japan) is an endoscopic visualisation technology which enhances the mucosal surface texture and underlying vasculature by utilising the concept that the wavelength of light determines the depth of penetration [54, 55]. Two modes, white light and NBI, are included in the system to provide real time noninvasive optical image enhancement of mucosa. Switching between the two modes is simply achieved by pressing a button on the video endoscope, camera head, or system processor [56]. In NBI mode, filters placed in front of the white light allow only blue light between 400 and 430 nm (centred at 415 nm) and green light between 525 and 555 nm (centred at 540 nm) to be emitted simultaneously. Blood vessels in the superficial mucosa appear brown as the blue light penetrates shallowly and corresponds to the peak absorption spectrum of haemoglobin. Conversely, the green light penetrates deeper to highlight thicker blood vessels in the submucosa, and these vessels appear cyan [54, 55]. Reflected light is captured by a charge coupled device (CCD) located at the tip of the endoscope and is reconstructed by an image processor into a coloured composite image that is then displayed on a high-definition monitor screen [56]. In addition to the excellent resolution that can be maintained up to 2 mm away from the mucosa due to the physical zoom property, further enhancement of the mucosal texture and microvascular structures is possible with magnifying endoscopy [55, 57]. Up to 80 times optical magnification is available with the 2-band red-green-blue sequential NBI systems (e.g., Evis Lucera 260 Spectrum), whereas 1.2 times digital zoom and 1.5 times digital zoom are available with the coloured CCD systems (e.g., Evis Exera II and Evis Exera III).

As previously stated, potentially malignant and malignant lesions have distinct microvascular morphology as angiogenesis is an early occurrence in carcinogenesis [58–60]. Neoplastic lesions appear as areas with scattered spots with a well-demarcated border, and can therefore be differentiated from inflammatory lesions which have an ill-demarcated border [61, 62]. These brown spots represent superficial vessels such as the intrapapillary capillary loops (IPCL). Visualisation of the vasculature gives clinicians a better idea of the true extent of lesions, and can therefore guide the position of biopsy and resection margins [61, 63, 64]. Furthermore, changes in the degree of dilation, meandering, tortuosity, and calibre of IPCLs indicate the severity of pathology present [58, 59, 61].

**Figure 3:**
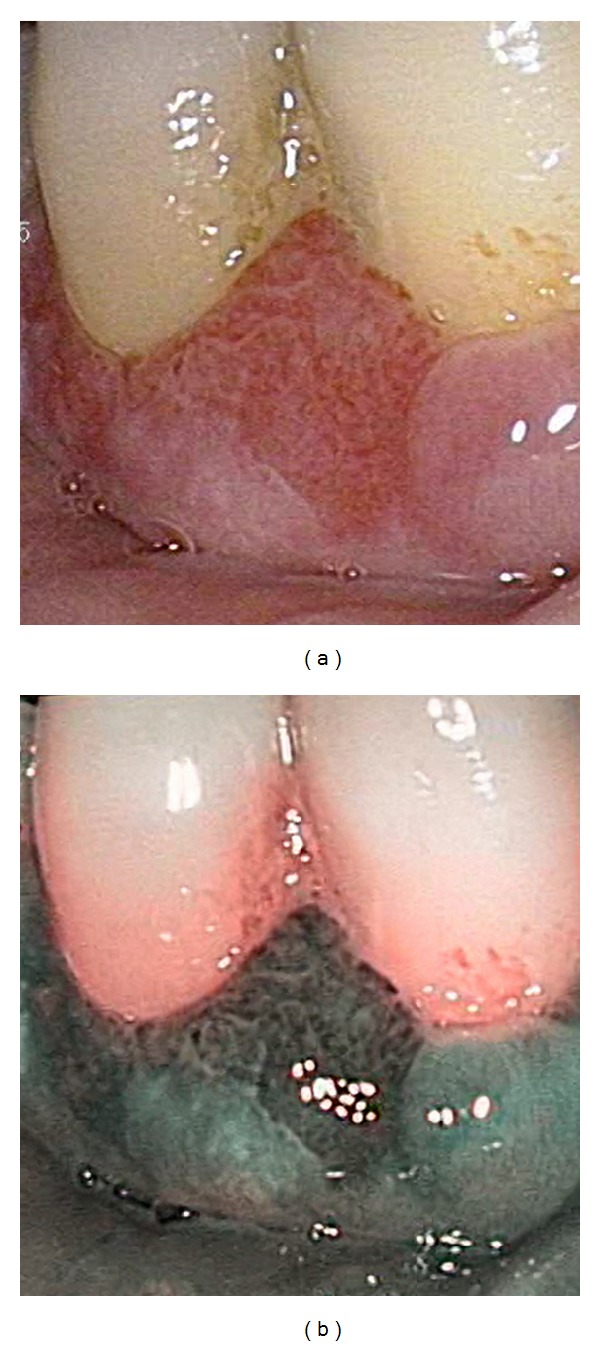
SCC of the gingiva viewed with endoscopic white light (a). Same lesion viewed in NBI mode demonstrating Type IV IPCL pattern (b).

Oral lesions may be classified using Takano et al.'s [61] IPCL classification for oral mucosa based on the most advanced IPCL pattern present. Type I IPCL pattern is characterised by regular brown dots when loops are perpendicular to the mucosa or waved lines when parallel [61]. Although Type I IPCL pattern is typically associated with normal mucosa [61], a study by Yang et al. [65] involving 154 patients with newly diagnosed leukoplakia reported 17% frequency of dysplasia in lesions displaying this pattern. Therefore, clinicians should still retain a degree of suspicion and use clinical judgement when examining leukoplakia with Type I IPCL pattern. By contrast, Type II which has dilated and crossing IPCLs, and Type III which displays elongated and meandering IPCLs [61] are more frequently associated with dysplasia [65, 66]. While Type II is usually associated with nonneoplastic and inflammatory lesions, Yang et al. [65] reported 92% frequency of dysplasia for leukoplakia with this pattern, and Type III IPCL pattern had 100% frequency of dysplasia. These findings are supported by another study [66] which described similar classes of microvascular patterns. In this study [66], the IPCL Types II and III equivalents were associated with OPMDs and carcinoma. Type IV IPCL pattern, however, is indicative of neoplasia [65] and is characterised by large vessels IPCL pattern destruction and angiogenesis (Figure 3) [61]. Any lesion with Types III and IV should therefore be biopsied [67], particularly since the use of Types III and IV as the criteria for differentiating high-grade dysplasia, CIS, and invasive carcinoma from normal mucosa has been shown to have 85% sensitivity, 95% specificity, 74% positive predictive value (PPV), 97% negative predictive value (NPV), and 93% accuracy (Table 3) [65].

The efficacy of NBI primarily depends on light penetrating the epithelium to enhance the vasculature. A study by Lin et al. [70] found that areas with nonkeratinized thin stratified squamous epithelium had a significantly higher prevalence of brownish spots than areas with keratinized epithelium or epithelium thicker than 500 *μ*m. However, Yang et al. [65] reported that the degree of keratinization did not affect visualisation of the underlying vasculature unless hyperkeratosis associated with leukoplakia was present. Visualisation of the microvasculature is possible through thin homogenous leukoplakia, but the vasculature will appear vague, blurry, or be completely obstructed where there is thick homogenous leukoplakia [67]. In the latter case, the IPCL pattern of the surrounding mucosa is often observed to guide the determination of the lesion's IPCL class; however, this is not completely reliable as one study [67] found dysplasia in 28% of thick homogenous leukoplakia surrounded by IPCL Type I. Instead, the degree of hyperkeratinization may be indicative of the degree of dysplasia, as IPCL Type I was only found beneath thin homogenous leukoplakia, whereas Types II and III were observed around thick homogenous leukoplakia.

To date, there are only a few papers that have evaluated the use of NBI in just the oral cavity. The sensitivity, specificity, PPV, NPV, and accuracy for detecting oral neoplasia with NBI ranged from 95 to 96%, 97 to 100%, 91 to 100%, and 93 to 99% and 97%, respectively (Table 3) [68, 69]. In comparison, the ranges for white light were generally lower at 51 to 64%, 96 to 100%, 82 to 100%, 87 to 90%, and 68 to 89%, respectively. Chronic inflammation and chronic post-radiotherapy changes contributed to false positives, and this may be compounded by operator inexperience in recognising the different IPCL changes associated with inflammatory and neoplastic lesions. Regardless, NBI has great potential as a valuable adjunct to COE as it can detect malignancies that might otherwise be missed with white light. 

There is currently no published clinical trial evaluating the efficacy of NBI for specifically detecting OPMDs in patients without oral cancer. Nonetheless, Nguyen et al. [72] conducted a prospective study involving 73 patients with head and neck cancer and found that the sensitivity for detecting moderate dysplasia or worse was at 96% with NBI, which was better than white light which had only 38%. It is possible that the efficacy for detecting dysplasia in OPMDs with NBI will be similar to that noted for OSCCs.

Although the use of NBI as an adjunct to COE for detecting OMPDs and OSCCs shows promise, the literature is still very limited. Results from published papers cannot be generalised to the general population as all studies have been conducted in specialist settings. In addition, NBI is only intended for use in secondary and tertiary settings due to the cost of the technology and training required. More prospective clinical trials are required to evaluate the efficacy of NBI for aiding the detection and surveillance of OPMDs and OSCCs.

## 5. Limitations of Optical Aids

The literature indicates that optical aids are effective in highlighting oral mucosal abnormalities but cannot effectively differentiate between those which are considered “low risk” or “high risk.” With VELscope and Identafi, this can be attributed to the biological basis which contributes to LAF of the oral mucosa. As previously discussed, LAF is a product of alterations in the metabolic activity in the epithelium and changes to the collagen architecture in the stroma [27]. Haemoglobin also has a significant influence on autofluorescence spectra of malignancies [73]. During oncogenesis, there is increased cellular proliferation resulting in increased metabolic activity combined with architectural changes in the stroma and angiogenesis, all of which contribute to LAF [27, 29, 50–52]. Benign inflammatory lesions such as oral lichen planus display increased vascularity and inflammation which introduces haemoglobin into the tissues and thus contributes to LAF. Vascular lesions also display LAF due to the increase in local haemoglobin content in the vasculature. In addition to this, not all dysplasias, particularly those at an early stage, display LAF [19]. Clinically, it cannot be determined if LAF is due to neoplasia or to benign inflammatory origin; therefore, autofluorescence alone cannot discriminate between “high-risk” and “low-risk” lesions, and careful clinical correlation is required. A similar dilemma exists for NBI as use of the technology is primarily based on clinically analysing the morphology of the underlying microvasculature. While applying a defined classification system is beneficial for interpreting IPCL patterns, it is still a subjective method and is not infallible as each class of IPCL pattern does not always correspond to a particular histopathological diagnosis [65]. Further complicating the matter is keratosis, which prevents clear visualisation of the underlying microvasculature [67]. Abnormal microvasculature patterns associated with chronic inflammation or vascular lesions can also act as confounders when using NBI [69]. Therefore, while these visualisation adjuncts can demonstrate the presence of an abnormality, they cannot effectively differentiate between “high-risk” and “low-risk” lesions in their current state.

## 6. Other Approaches to Optical Imaging

### 6.1. Autofluorescence with Endoscopic Techniques

In order to improve the accuracy of optical imaging techniques, different technologies have been combined into multimodal imaging devices. One example is the combination of autofluorescence imaging with a high-resolution microendoscope system (HRME). While autofluorescence can be used for examination of a wide field, HRME is designed to assess specific sites. Pierce et al. [74] found increased sensitivity and specificity for the detection of OED and OSCC by combining autofluorescence imaging and a high-resolution microendoscope system compared to either technology alone.

Another example is the use of NBI with fluorescence imaging. A prospective study by Nguyen et al. [72] assessed the use of autofluorescence and NBI on patients with a history of SCC in the head and neck. Sensitivity for detecting dysplasia was higher with autofluorescence (96%) and NBI (96%) than with white light (37%), and high specificity was noted for the combined use of autofluorescence and NBI. The addition of autofluorescence and NBI to white light endoscopic examination also influenced the management of 6% of patients. Similar results were reported in a study that assessed for dysplasia in patients with Barrett's oesophagus using the combination of fluorescence imaging, reflectance, and light scattering microscopy. In this study, the sensitivity and specificity were 93% and 100%, respectively, when at least two of the three lights indicated neoplasia [75]. The addition of NBI following autofluorescence imaging in the surveillance of Barrett's oesophagus also reduced the false-positive rate of autofluorescence from 40% to 10% without affecting sensitivity [76]. 

An alternative combination commercialised for use in the oesophagus is the endoscopic trimodal imaging (ETMI) system which incorporates white light endoscopy, autofluorescence imaging, and NBI. As with the previous study, the use of NBI following autofluorescence imaging removed some false-positive findings; however, it also misclassified areas of neoplasia [77]. While ETMI is more effective than targeted biopsies in the surveillance of Barrett's oesophagus, the overall histological yield is greater through the use of standard video endoscopy with both random and targeted biopsies [77].

### 6.2. Fluorescence Lifetime Imaging

Another potentially useful technology is fluorescence lifetime imaging, which assesses the decay of fluorescence [78, 79]. Following excitation, autofluorescence is emitted for up to ten nanoseconds and during this time, the decay in fluorescence can be measured [80]. It is suggested that fluorescence lifetime imaging is unaffected by excitation intensity, fluorophore concentrations, or attenuation due to tissue absorption or scattering [79]. Although laser guided fluorescence provides promise as a diagnostic tool that can act as an “optical biopsy”, it is impractical for screening purposes where wide-field autofluorescence visualisation is desirable. Consequently, Galletly et al. [79] devised a method using fluorescence lifetime imaging which allows for a large field of view. Therefore, this could be used for both detecting small or poorly visible lesions and for delineating surgical margins for excision [79].

In the oral cavity, OPMDs are associated with increased fluorescence decay times [81]. This model utilised a 410 nm wavelength and measured emission at the 633 nm wavelength and much like previous studies calculated a threshold value to classify lesions. Chen et al. [81] were able to accurately distinguish cases of verrucous hyperplasia and OED from normal mucosa using this approach.

### 6.3. Optical Coherence Tomography

Optical coherence tomography (OCT) is an optical imaging modality which utilises low-power infrared light between 750 and 1300 nm and a Michelson interferometer to produce high-resolution, cross-sectional, and subsurface tomographic images of tissue microstructure [82, 83]. Interaction of the light with the tissue surface causes scattering, and images are generated by measuring the echo time delay and the intensity of back-scattered light [84, 85]. The depth of penetration for OCT imaging is approximately 1 to 3 mm depending on the tissue structure, depth of focus of the probe used, and pressure applied to the tissue surface [86]. 

OCT has been profoundly used in ophthalmic practice to provide *in vivo* “optical biopsy” of the retina [87], in addition to dermatological applications which include evaluation of skin tumours [88], and inflammatory disease [89].

OCT can be used to identify architectural changes in the keratin cell layer, epithelial layer, basement membrane, lamina propria, and rete pegs of oral mucosa [90]. Although OCT is capable of assessing lesions for neoplastic changes by determining the thickness of the epithelial layer, integrity of the basement membrane, and changes in the lamina propria [91], it is still incapable of providing enough cellular information to grade OPMDs [90]. Early research revealed that while OCT imaging displayed 93% sensitivity and 97% specificity for diagnosing OSCCs when compared to histology in one study [92], another study reported that its ability to differentiate between different oral mucosal abnormalities was poor [91]. Recent research reflects the latter finding, with sensitivity and specificity of 85% and 78%, respectively, for the identification of OPMDs using *ex vivo* biopsies [93]. Tsai et al. have analysed OCT profiles for the delineation of OSCC margins and suggested that this could be used to develop an algorithm for the detection and delineation of OSCCs [94, 95]. Further research is required on the potential application of OCT to improve and define excisional margins during surgical management of OPMDs and OSCCs.

### 6.4. Angle-Resolved Low-Coherence Interferometry (a/LCI)

Angle-resolved low-coherence interferometry (a/LCI) is another noninvasive optical spectroscopic technique which, like laser induced autofluorescence and OCT, can be used to perform an “optical biopsy” of intact, living tissue [96, 97]. a/LCI utilises measurements of angular light scattering of cellular nuclei to calculate the average nuclear diameter, and like OCT, this method can provide information as a function of depth of epithelium [96, 97]. The detailed science behind a/LCI has been covered elsewhere [97]. 

Differences in the average nuclear diameter can be used to differentiate dysplasia from normal tissue, as increased nuclear diameter is associated with neoplastic progression [98–101]. Wax et al. [100] created an algorithm for the use of a/LCI by calculating threshold values for dysplasia with a rat oesophageal carcinoma model, and on prospective analysis, this technique proved to be highly accurate with sensitivity and specificity of 91% and 97%, respectively [101]. The advantage of this technology over other technologies such as autofluorescence or NBI is that it allows for targeted sampling through depth sensitivity analysis. Using a/LCI on human biopsy tissue, Brown et al. [102] confirmed that analysing deeper tissue segments near the basal layer, when compared to more superficial layers, provided the highest degree of accuracy for observing dysplasia and hence had the greatest diagnostic potential. 

Zhu et al. [103] created a prototype a/LCI system which was programmed to assess, through an endoscope, the mean nuclear diameter of oesophageal tissue cells located at 200 to 300 *μ*m in depth, near the basal layer. Forty-six patients undergoing routine endoscopic screening for Barrett's oesophagus were assessed using this prototype system to obtain an “optical biopsy” which was then compared with histology [98]. The determined threshold value for this model had 100% sensitivity, and specificity of 84% when all samples were included and 85% when only samples with Barrett's oesophagus were considered [98]. Terry et al. [104] found that a/LCI was able to diagnose dysplasia on *ex vivo *samples of colonic tissue with 85% accuracy and suggested that this technique may be ready for *in vivo *studies for the diagnosis of intestinal dysplasia. 

While research is at an early stage, a/LCI appears to be a highly accurate method of diagnosing dysplasia during routine screening of patients with Barrett's oesophagus. Larger-scale trials are required to determine the most appropriate threshold value and whether this technology can replace the need for physical biopsies. There are currently no published papers regarding the use of a/LCI in the oral cavity; however, the use of this technology may reduce the number of physical biopsies through its potential ability to provide on-the-spot diagnosis of lesions.

### 6.5. Use of Algorithms

As previously discussed, a severe limitation of existing optical imaging techniques is the arbitrary nature of interpretation leading to a high level of interoperator variability. To reduce this, a newer approach targeted towards creating algorithms based on quantitative data allows for computer analysis of the fluorescence properties and thus removes operator bias. Such models have been established with a high degree of accuracy for the diagnosis of oesophageal cancer using laser-induced autofluorescence at 410 nm excitation wavelength [105–107]. Vo-Dinh et al. found that at this excitation wavelength, oesophageal malignancies were associated with reduced fluorescence intensity and changes in fluorescence spectra [105, 106]. While malignancies were associated with less fluorescence intensity, the intensity was found to be an inconsistent parameter and a more independent measurement was required. Instead, the group assessed the fluorescence spectra of normal and malignant tissue between 430 and 720 nm and normalized for intensity to remove variations between lesions. The most significant differences in differential normalized fluorescence (DNF) in malignancies were found at 480 nm and 660 nm wavelengths [105, 106]. Initial evaluation found this model could differentiate malignant tissue from normal tissue with greater than 98% accuracy [105, 106]. Such an approach removes the operator from the decision-making process and allows for simple computer-based classification of lesions into benign and malignant. Due to the high accuracy of this technique, it may be used as an “optical biopsy”, thereby significantly reducing the need for a physical biopsy and the associated morbidity, time, and financial costs [106]. When this technique using DNF data was extended to assess for high-grade dysplasia in Barrett's oesophagus, it was associated with a high specificity and a high sensitivity [108]. In this study however, low-grade dysplasia was considered benign as no surgical intervention was required. Of concern, only 28% of low-grade with focal high-grade dysplasias were considered positive [108]. This may be due to the endoscopic laser only being able to assess small areas of tissue at a time, while the biopsy sample may include adjacent tissue with foci of high-grade dysplasia. Unlike direct tissue fluorescence visualization, the presence of inflammation does not create false-positive findings using DNF indices [109]. A similar approach could be utilised in the oral cavity to increase the sensitivity of autofluorescence visualisation. If molecular markers associated with LAF could be found, it is possible to target these by identifying their associated fluorescence spectra.

To reduce the effect of operator bias, Roblyer et al. [28] introduced an objective method of distinguishing oral neoplasia from benign tissue using autofluorescence with a 405 nm light source. An algorithm was created on a test group of patients presenting with oral mucosal lesions as well as volunteers with healthy mucosa. The authors found a reduction in green fluorescence and an increase in red fluorescence to be highly associated with neoplastic tissue. They determined that the normalized red-to-green ratio was able to predict the risk of neoplasia and calculated a threshold value beyond which the area would be considered suspicious for neoplasia. Using the images obtained under 405 nm excitation, a probability map was generated by calculating the risk of neoplasia for each area of tissue, and areas with a risk of dysplasia being greater than 50% were highlighted. In the training group, this algorithm was associated with a sensitivity and specificity of 96%, whereas in the validation group, these values were 100% and 91%, respectively. As with the approach taken in oesophageal cancers, this method uses direct tissue autofluorescence but removes the factor of operator bias. Future research should examine the construction of a simple device which could create similar probability maps chairside, as this would improve the usefulness of direct tissue fluorescence in helping determine biopsy location and surgical margin delineation.

## 7. Conclusions

Detection of OPMDs before they advance to OSCC is predicted to improve survival rates for oral cancer. Evidence indicates that COE is a poor discriminator of oral mucosal lesions, and this has led to the development of several adjunctive visualisation aids. VELscope is associated with high sensitivity and can assist in the detection of additional lesions; however, additional research is required to reduce the incidence of false positives. Further research is also required to assess the efficacy of Identafi, a multispectral device with limited available scientific and clinical literature. NBI shows great promise as a useful adjunct to COE, as several studies have reported that it performed better than white light at detecting malignancies. However, there is currently no published clinical trial evaluating the efficacy of NBI for detecting OPMDs in patients who do not have confirmed oral cancer. Therefore, before these visualisation adjuncts can gain widespread use, larger well-designed prospective studies for each technology are required. Further studies are also required into the molecular basis for fluorescence imaging to help determine the factors contributing to false-positive and false-negative findings. Research into the biological mechanisms of angiogenesis associated with oral cancer will hopefully provide a clearer understanding of the microvascular changes that occur in OPMDs and OSCCs. This in turn may lead to more effective and predictable methods for accurately interpreting NBI data. Future approaches to optical imaging could involve real time quantitative evaluation to determine a diagnosis for oral mucosal lesions rather than simply highlighting the presence of abnormalities, thus, making the possibility of “optical biopsy” a clinical reality.

## Figures and Tables

**Table 1:** Published papers on the use of VELscope in specialist practice.

Purpose	Paper	Type of study	Sample population	Sensitivity (%)	Specificity (%)	PPV (%)	NPV (%)	Accuracy (%)	Notes
Used to detect OPMDs only	Poh et al. [17]	Case report	3 case reports of patients with a history of either oral dysplasia or CIS.	—	—	—	—	—	All cases demonstrated LAF in the area where there was a lesion. 1 patient had moderate epithelial dysplasia, another had CIS, and the third case had both severe dysplasia and CIS.
	Awan et al. [18]	Prospective cohort study	126 patients with oral white or red lesions suspicious of OPMD.	84	15	38	61	—	Of the 126 lesions, 7 dysplasias had no LAF and 61 nondysplastic lesions had LAF.
	Farah et al. [19]	Prospective cohort study	112 patients with white or mixed red-white lesions suspicious of OPMD.	30	63	19	75	55	Interpretation based on VELscope findings only.
				46	68	29	82	63	Interpretation based on both COE and VELscope findings.
	Rana et al. [20]	Cross-sectional study	289 patients in total with an OPMD (166 patients examined with COE, 123 patients examined with both COE and VELscope).	100	74	—	—	—	VELscope had higher sensitivity (100% versus 17%) but lower specificity (74% versus 97%) when compared to COE.

Used to detect OPMDs and/or oral cancer	Lane et al. [21]	Prospective cohort study	44 patients with biopsy-confirmed oral dysplasia or OSCC.	98	100	100	86	—	91% of severe dysplasia and CIS showed LAF. 100% of OSCCs had LAF.
	Mehrotra et al. [22]	Cross-sectional study	258 patients in total with clinically innocuous lesions (102 patients were examined with ViziLite; 156 patients were examined with VELscope).	50	39	6	90	—	6 dysplasias did not display LAF. VELscope did not detect any additional lesions following COE.
	Koch et al. [23]	Prospective cohort study	78 patients with clinically diagnosed SCC or suspicious epithelial lesion.	97	96	94	98	—	For diagnosing SCC only.
				94	98	97	96	—	For diagnosing SCC/dysplasia.
	Scheer et al. [24]	Prospective cohort study	64 patients referred to specialist clinic to rule out OSCC.	100	81	55	100	—	False-positive rate of 15.6%. LAF significantly associated with dysplasia or invasive carcinoma when compared to normal tissue (*P* < 0.0001).

Used to detect oral cancer only	Poh et al. [25]	Prospective cohort study	20 consecutive patients with biopsy-confirmed oral cancer.	—	—	—	—	—	All tumours showed LAF, with a significant correlation between high-grade dysplasia and LAF (*P* < 0.0001). 95% (19/20) of tumours had VELscope margins that extended beyond the clinically visible tumour.

OPMD: oral potentially malignant disorder; CIS: carcinoma in situ; LAF: loss of autofluorescence; COE: conventional oral examination; OSCC: oral squamous cell carcinoma.

**Table 2:** Published papers on the use of VELscope in general practice.

Purpose	Paper	Type of study	Sample population	Sensitivity (%)	Specificity (%)	PPV (%)	NPV (%)	Accuracy (%)	Notes
Used to detect OPMDs and/or oral cancer	Huff et al. [41]	Parallel cohort study	959 patients presenting to a private practice over a 12-month period received COE only. 905 presenting to the same private practice over a separate 12-month period received COE and VELscope examination.	—	—	—	—	—	For the COE only cohort, there was a 0.83% prevalence of mucosal abnormalities, with none being potentially malignant. For the combined COE and VELscope examination cohort, there was a 1.3% prevalence of mucosal abnormalities, with 83% of these being potentially malignant.
	Truelove et al. [42]	Prospective cohort study	620 patients seeking routine or emergency dental treatment at a dental school received both COE and VELscope examination.	—	—	—	—	—	Patients initially examined by dental students before the attending faculty. 5 dysplasias detected with VELscope were not found by COE.
	McNamara et al. [43]	Prospective cohort study	130 consecutive patients presenting to a screening clinic for routine dental care received both COE and VELscope examination.	67*	6*	6*	67*	—	No abnormalities detected with VELscope that were not found by COE. 93.8% (30/32) false-positive rate. 1 false-negative case. VELscope findings statistically different from scalpel biopsy gold standard (*P* = 0.0001).

*Calculated based on provided values.

OPMDs: oral potentially malignant disorders; COE: conventional oral examination.

**Table 3:** Published papers on the use of NBI in the oral cavity.

Paper	Purpose	Type of study	Sample population	Sensitivity (%)	Specificity (%)	PPV (%)	NPV (%)	Accuracy (%)	Notes
Katada et al. [63]	Used to detect superficial SCC	Case report	2 patients with oesophageal cancer.	—	—	—	—	—	Coincidental finding of OSCC at floor of mouth.

Piazza et al. [68]	Used to detect dysplasia and SCC	Prospective cohort study	96 patients with biopsy-confirmed or previous-treated OSCC or oropharyngeal SCC.	96	100	100	93	97	Combined the use of NBI with high-definition television. 27% (26 of 96) patients had a diagnostic benefit with the use of NBI and high-definition television.

Takano et al. [61]	Investigated the types of IPCL patterns	Prospective cohort study	41 patients with normal mucosa, or nonneoplastic or neoplastic lesions.	—	—	—	—	—	Devised the IPCL classification of oral mucosa.

Chu et al. [69]	Used to detect dysplasia and OSCC	Prospective cohort study	101 patients with treated OSCC.	95%	97%	91%	99%	97%	Had difficulty diagnosing hyperkeratotic lesions, tumours at the tongue base, and recurring tumours. Chronic inflammation, postoperative radiation, colouration, or mucosal staining interfered with diagnosis.

Lin et al. [70]	Investigated the visibility of brownish spots in different types of epithelium	Prospective cohort study	125 patients with CIS or SCC in the head and neck.	—	—	—	—	—	Areas with nonkeratinized thin stratified squamous epithelium had a significantly higher prevalence of brownish spots than areas with keratinized epithelium or epithelium thicker than 500 *μ*m.

Tan et al. [64]	Used NBI to influence management of oral erythroplakia	Case report	1 patient with erythroplakia.	—	—	—	—	—	NBI was used to determine resection margins, which were beyond the clinically visible margins.

Yang et al. [65]	Correlated NBI clinical findings with histopathology	Retrospective cohort study	154 patients with oral leukoplakia.	—	—	—	—	—	The IPCL classification had a significant statistical association with the severity of pathology.

Yang et al. [67]	Evaluated the use of NBI for assessing and managing oral leukoplakia	Retrospective cohort study	160 patients with clinical homogenous oral leukoplakia.	—	—	—	—	—	All cases of thin leukoplakia had IPCL Type I and were confirmed as squamous hyperplasia. Thick leukoplakia had IPCL Type I, II, or III, and a significant correlation between pathology and NBI images was present (*P* < 0.0001).

Yang et al. [71]	Used IPCL patterns made visible by NBI to diagnose high-grade dysplasia, CIS, and OSCC	Retrospective case-control study	414 patients with oral leukoplak.ia	15%	60%	7%	79%	53%	Criteria used was “brownish spots and demarcation line with irregular microvascular pattern”.
				77%	55%	24%	93%	58%	Criteria used was “well-demarcated brownish area with thick dark spots and/or winding vessels”.
				85%	95%	75%	97%	93%	Criteria used was “the intraepithelial papillary capillary loop (IPCL) Type III … and Type IV”.

Yang et al. [66]	Investigated the IPCL morphology of OSCC and correlated the pattern with infiltration depth and disease severity	Retrospective cohort study	80 patients with-biopsy confirmed OSCC	—	—	—	—	—	The IPCL pattern moved from tortuous and dilated to twisted and elongated to angiogenesis and destruction of IPCL as the severity of OSCC increased. Depth of infiltration increased with the degree of severity.

Nguyen et al. [72]	Compared white light, autofluorescence, and NBI to detect moderate dysplasia or worse	Prospective cohort study	73 patients with known or treated head and neck SCC	96	79	85	94	—	Autofluorescence and NBI were significantly more sensitive than white light. NBI and white light were more specific than autofluorescence.

NBI: narrow band imaging; OSCC: oral squamous cell carcinoma; SCC: squamous cell carcinoma; IPCL: intrapapillary capillary loop; and CIS: carcinoma in situ.
